# Effects of Sirolimus treatment on patients with β‐Thalassemia: Lymphocyte immunophenotype and biological activity of memory CD4 ^+^ and CD8 ^+^ T cells

**DOI:** 10.1111/jcmm.17655

**Published:** 2023-01-10

**Authors:** Matteo Zurlo, Francesco Nicoli, Davide Proietto, Beatrice Dallan, Cristina Zuccato, Lucia Carmela Cosenza, Jessica Gasparello, Chiara Papi, Elisabetta d'Aversa, Monica Borgatti, Chiara Scapoli, Alessia Finotti, Roberto Gambari

**Affiliations:** ^1^ Department of Life Sciences and Biotechnology, Section of Biochemistry and Molecular Biology University of Ferrara Ferrara Italy; ^2^ Department of Chemistry, Pharmaceutical and Agricultural Sciences University of Ferrara Ferrara Italy; ^3^ Department of Life Sciences and Biotechnology, Section of Biology and Evolution University of Ferrara Ferrara Italy; ^4^ Center Chiara Gemmo and Elio Zago for the Research on Thalassemia University of Ferrara Ferrara Italy

**Keywords:** autophagy, immunophenotype, memory T cell, mTOR, Sirolimus, β‐Thalassemia

## Abstract

Inhibitors of the mammalian target of rapamycin (mTOR) have been proposed to improve vaccine responses, especially in the elderly. Accordingly, testing mTOR inhibitors (such as Sirolimus) and other geroprotective drugs might be considered a key strategy to improve overall health resilience of aged populations. In this respect, Sirolimus (also known as rapamycin) is of great interest, in consideration of the fact that it is extensively used in routine therapy and in clinical studies for the treatment of several diseases. Recently, Sirolimus has been considered in laboratory and clinical studies aimed to find novel protocols for the therapy of hemoglobinopathies (e.g. β‐Thalassemia). The objective of the present study was to analyse the activity of CD4^+^ and CD8^+^ T cells in β‐Thalassemia patients treated with Sirolimus, taking advantages from the availability of cellular samples of the NCT03877809 clinical trial. The approach was to verify IFN‐γ releases following stimulation of peripheral blood mononuclear cells (PBMCs) to stimulatory CEF and CEFTA peptide pools, stimulatory for CD4^+^ and CD8^+^ T cells, respectively. The main results of the present study are that treatment of β‐Thalassemia patients with Sirolimus has a positive impact on the biological activity and number of memory CD4^+^ and CD8^+^ T cells releasing IFN‐γ following stimulation with antigenic stimuli present in immunological memory. These data are to our knowledge novel and in our opinion of interest, in consideration of the fact that β‐Thalassemia patients are considered prone to immune deficiency.

## 1 INTRODUCTION

Inhibitors of the mammalian target of rapamycin (mTOR) have been proposed by many studies to improve vaccine responses, especially in elderly. This was for instance reported to impact on the Flu vaccine efficacy.^1^, ^2^ Accordingly, testing mTOR inhibitors and other geroprotective drugs might be considered a key strategy to improve overall health resilience of aged populations.^3^, ^4^, ^5^ Among the effects of mTOR inhibitors responsible for improving the vaccine activity, enhanced CD8^+^ effector memory T‐cell function has been proposed.^6^, ^7^ Collectively, there is agreement on the fact that mTOR inhibitors might play an important role in boosting vaccination of elderly and fragile people as well as memory T‐cell function in general.^3^, ^4^, ^5^, ^8^, ^9^, ^10^

In this respect, Sirolimus (also known as rapamycin, a lipophilic macrolide isolated from a strain of *Streptomyces hygroscopicus*)^11^, ^12^ is of great interest, in consideration of the fact that it is extensively used in routine therapy and in clinical studies for the treatment of several diseases, such as renal, cardiac and liver transplantation,^13^, ^14^, ^15^, ^16^ systemic lupus erythematosus,^17^ lymphangioleiomyomatosis,^18^ tuberous sclerosis complex,^19^ recurrent meningioma,^20^ pancreatic neuroendocrine tumours,^21^ advanced differentiated thyroid cancers,^22^ advanced breast cancer,^23^ diffuse large B‐cell lymphomas^24^ and metastatic renal cell carcinoma.^25^

Recently, Sirolimus has been considered in laboratory and clinical studies aimed to find novel protocols for the therapy of hemoglobinopathies (e.g. β‐Thalassemia and sickle‐cell disease).^26^, ^27^, ^28^, ^29^, ^30^, ^31^ The β‐Thalassemias are due to more that 300 hereditary mutations of the β‐globin gene, inducing absence or low‐level synthesis of β‐globin in erythroid cells.^32^, ^33^, ^34^ It is widely accepted that high production of foetal haemoglobin (HbF) is beneficial for β‐Thalassemia patients.^35^, ^36^, ^37^, ^38^ In a recent study, we have reported biochemical, molecular and clinical results of the Sirolimus‐based NCT03877809 clinical trial (A Personalized Medicine Approach for β‐Thalassemia Transfusion Dependent Patients: Testing Sirolimus in a First Pilot Clinical Trial: Sirthalaclin).^39^, ^40^ The rationale of this trial was that Sirolimus is of interest in β‐Thalassemia, since it induces the expression of foetal haemoglobin (and this might contribute to ameliorate the clinical parameters of these patients), induces autophagy (thereby reducing the excess of free α‐globin) and, finally, might contribute to mobilization of erythroid cell from the bone marrow (thereby reducing anaemia).

The results of the trial were obtained in 8 patients with β^+^/β^+^ and β^+^/β^0^ genotypes, treated with a starting dosage of 1 mg/day Sirolimus for 24–48 weeks.^40^ The first finding of the study was that expression of γ‐globin mRNA was increased in blood and erythroid precursor cells isolated from β‐Thalassemia patients treated with low‐dose Sirolimus. A second important conclusion was that Sirolimus influences erythropoiesis and reduces biochemical markers associated with ineffective erythropoiesis (excess of free α‐globin chains, bilirubin, soluble transferrin receptor and ferritin). In most of the patients, a decrease of the transfusion demand index was observed. Altogether, the data obtained suggested that Sirolimus given at low doses modifies haematopoiesis and induces increased expression of γ‐globin genes in a subset of β‐Thalassemia patients.

The major end points of the NCT03877809 clinical trial were not aimed to explore changes in the functionality of memory T cells of Sirolimus‐treated patients.^39^ However, the possible effect of Sirolimus in β‐Thalassemia is intriguing, as recent reports have highlighted a high mortality rate in β‐Thalassemia patients affected by infectious bacterial and viral diseases,^41^, ^42^, ^43^, ^44^, ^45^, ^46^ probably due to co‐existing immune deficiencies.^47^, ^48^, ^49^ Immune dysfunctions characterizing thalassemia patients include changes in lymphocyte subsets, such as the accumulation of suppressor T cells and the reduced proliferative capacity and numbers of T helper cells, as well as the defective activity of natural killer (NK) cells. Similarly, an altered humoral immunity has been shown in patients with β‐Thalassemia.^48^ Therefore, efficient vaccination is of primary importance and molecules exhibiting boosting effects on vaccines should be considered of great relevance.

The objective of the present study was to analyse the activity of CD4^+^ and CD8^+^ T cells in β‐Thalassemia patients treated with Sirolimus, taking advantages from the availability of cellular samples from the NCT03877809 clinical trial. The approach was to verify IFN‐γ releases following stimulation of peripheral blood mononuclear cells (PBMCs) to CEF and CEFT peptide pools, that activate CD8^+^ and CD4^+^ T cells, respectively.

## 2 METHODS

### 2.1 Patient recruitment and treatment with Sirolimus

Recruitment of the Sirthalaclin pilot clinical trial and data collection (EudraCT n° 2018–001942‐33, NCT 03877809) was at the Thalassemia Centre of Azienda Ospedaliera‐Universitaria S.Anna, as extensively reported in Zuccato et al.^40^ All the patients were transfusion‐dependent (TDT, Transfusion Dependent Thalassemia). The β‐Thalassemia patients have been recruited among patients with β^+^/β^+^ and β^+^/β^0^ genotypes. The study was approved by the Ethical Committee in charge of human studies at Arcispedale S.Anna, Ferrara (release of the approval: 14 November 2018). The list of analysed patients is reported in Table 1.

**TABLE 1:** Characteristics of the nine transfusion‐dependent patients included in the study

Patient ID	Genotype	Age range (years)	Sex
1	β^0^39/β^+^IVSI‐110	56–60	Female
2	β^0^39/β^+^IVSI‐110	46–50	Female
3	β^0^39/β^+^IVSI‐110	40–45	Female
4	β^0^39/β^+^IVSI‐110	40–45	Female
5	β^0^39/β^+^IVSI‐6	46–50	Female
6	β^0^39/β^+^IVSI‐110	40–45	Male
7	β^+^IVSI‐110/ β^+^IVSI‐110	40–45	Male
8	β^0^39/β^+^IVSI‐110	50–55	Female
9	β^0^39/β^+^IVSI‐110	46–50	Male

The investigational drug in the form of coated tablets (0.5 mg Sirolimus) has been provided to the patients in adequately labelled blisters. The starting Sirolimus dosage was 1 mg/day. All other standard treatments, including blood transfusions and iron chelation therapy, have been continued, in agreement with the International Thalassemia Federation (T.I.F.) Guidelines.^50^ The analysis of the Sirolimus blood content in treated thalassemia patients was carried out as described in detail by Zuccato et al.^40^

### 2.2 Analysis of the immunophenotype

Peripheral blood mononuclear cells (PBMCs) were isolated from whole blood before drug administration (Day 0) and in different time‐points during treatment (Day 90 and Day 180). PBMCs were obtained by density centrifugation on Lympholyte (Cedarlane, Burlington, Canada) following manufacturer instruction, washed in PBS and frozen in standard FBS/10% DMSO freezing medium. For analysis, cells were thawed in RPMI‐1640 medium and washed in PBS (Lonza); the staining was performed using 1 million cells for each time point. The cells were firstly stained with LIVE/Dead™ Fixable Aqua—Dead Cell Stain Kit (Thermo Fisher) and incubated 10′ in the dark. After a PBS wash, cells were stained with a mixture of antibodies (see Table 2) targeting different membrane receptors (antibodies against CD3, CD4, CD8, CD14, CD19 and CD25) and incubated 15′ in the dark. After an additional PBS wash, cells were permeabilized using eBioscience Foxp3/Transcription Factor Staining Buffer Set (Invitrogen by Thermo Fisher) and stained with the last antibody against FoxP3 transcription factor. At the end of the staining process, cells were washed in PBS to reduce the background, resuspended in 200 μl of PBS and analysed by flow cytometry using the BD FACSCanto II cell analyser (Becton Dickinson) as previously reported.^40^

**TABLE 2:** List of antibodies employed for Lymphocyte immunophenotyping

Ab	Fluorochrome	Cat.n.	Manufacturer
CD19	PacificBlue	302232	BioLegend
FOXP3	AlexaFluor488	53‐4776‐42	Invitrogen by Thermo Fisher
CD25	PE	12‐0259‐42	Invitrogen by Thermo Fisher
CD3	PerCP	300428	BioLegend
CD14	PE‐Cy7	25‐0149‐42	Invitrogen by Thermo Fisher
CD4	APC	300514	BioLegend
CD8	APC‐Cy7	557834	BD Pharmingen

### 2.3 Autophagy detection in memory T cells subpopulations

PBMCs were treated as above mentioned in the analysis of the immunophenotype section and stained with an autophagosome tracker (Cyto‐ID) and a different antibody mix in order to detect autophagy levels in different memory T cells subpopulations inside CD4^+^ and CD8^+^ lymphocytes (see Table 3). Cells were stained with Cyto‐ID autophagy detection kit 2.0 (Enzo LifeSciences) following manufacturer protocol and then washed in PBS and stained with LIVE/Dead™ Fixable Aqua—Dead Cell Stain Kit and incubated 10′ in the dark. After an additional wash step, cells were stained with the antibody mix showed in Table 3 and incubated 15′ in the dark. Finally, cells were washed in PBS, resuspended in 200 μl of PBS and analysed by flow cytometry using the BD FACSCanto II cell analyser as elsewhere reported.^51^, ^52^

**TABLE 3:** List of antibodies employed for T memory Lymphocyte subset typing

Ab	Fluorochrome	Cat.n.	Manufacturer
CD8	APC‐Cy7	557834	BD Pharmingen
CD45RA	PerCP‐Cy5.5	45‐0458‐42	Invitrogen by Thermo Fisher
CD27	APC	130‐113‐626	Miltenyi Biotec
CD4	eFluor450	4331794	Invitrogen by Thermo Fisher
CCR7	PE‐Cy7	557648	BD Pharmingen

### 2.4 ELISPOT for characterization of CD8+ and CD4+ T memory cells functionality

Elispot assays were performed using the Human IFN‐γ ELISpot PLUS (HRP) kit (MabTech) with precoated plates. Thawed PBMCs were diluted in RPMI 1640 medium containing 10% foetal bovine serum (FBS; Biowest) and incubated for 24 h at 37°C in humidified 5% CO2 atmosphere. After 24 h, cells were counted and seeded (25 × 10^3^ cells per well for CEF stimuli and 100 × 10^3^ cells per well for CEFT stimuli) in duplicate in 96‐well precoated plate (Mabtech) and stimulated with ProMix™ CEF and CEFT Peptide Pools (ProImmune); cells of each tested donor were also incubated with medium alone (negative control) and with an anti‐CD3 monoclonal antibody (Mabtech) as a positive control of stimulation. Plates were incubated for 24 h, processed according to the manufacturer's instruction, acquired with Eli. Expert ELISPOT automated reading system (A.EL.VIS) and analysed with Eli.Analyse software. The number of specific IFNγ‐secreting T cells, expressed as spot‐forming units (SFU) per million cells, was calculated by subtracting the negative control values.^52^

### 2.5 Analysis of cytokines, chemokines and growth factors

Plasma samples were isolated from β‐Thalassemia patients by direct centrifugation of the whole blood, as described in Zuccato et al.^40^ Proteins were measured using Bio‐Plex Human Cytokine 27‐plex Assay (Bio‐Rad) as suggested by the manufacturer and described in Gasparello et al.^53^ and in the Supplementary Materials (SM1). The assay allows the multiplexed quantitative measurement of 27 cytokines/chemokines (including FGF basic, Eotaxin, G‐CSF, GM‐CSF, IFN‐γ, IL‐1β, IL‐1rα, IL‐2, IL‐4, IL‐5, IL‐6, IL‐7, IL‐8, IL‐9, IL‐10, IL‐12, IL‐13, IL‐15, IL‐17A, IP‐10, MCP‐1, MIP‐1α, MIP‐1β, PDGF‐BB, RANTES, TNF‐α and VEGF) in a single well. Briefly, an amount of 50 μl of cytokine standards and plasma samples were incubated with 50 μl of anti‐cytokine conjugated beads in a 96‐well plate. After multiple washing, 25 μl of diluted detection antibody were added to each well and the plate was incubated for 30 min at room temperature with shaking. After washing, 50 μl of streptavidin‐phycoerythrin was added, and the plate was incubated with shaking at room temperature, washed and read using the Bio‐Plex 200 array reader (Bio‐Rad). Data were analysed by the Bio‐Plex Manager Software (Bio‐Rad).^53^

### 2.6 Statistical analysis

Unless otherwise stated, all the data were normally distributed and presented as mean ± S.D. Statistical differences between groups were compared using Wilcoxon test or one‐way anova (analyses of variance between groups) for repeated measures followed by Dunnet post hoc tests. Statistical differences were considered significant when *p* < .05 (*), highly significant when *p* < .01 (**).

## 3 RESULTS

### 3.1 Lymphocyte immunophenotyping of Sirolimus‐treated β‐Thalassemia patients

**FIGURE 1:**
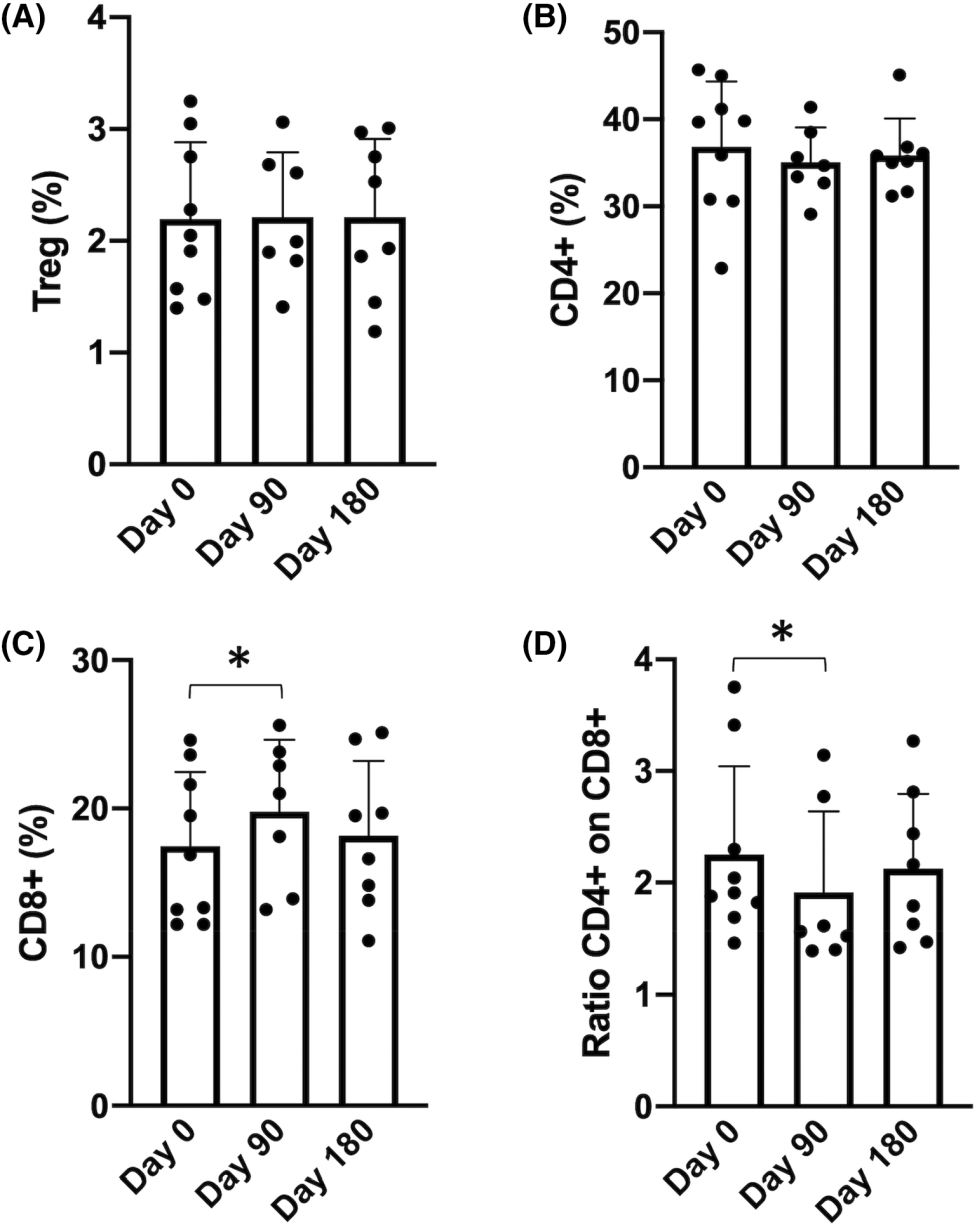
Summary representing the % of regulatory T lymphocytes (Treg) in respect to the total number of CD4^+^ T lymphocytes (A). (B–C) Summary representing the % of CD4^+^ and CD8^+^ T lymphocytes, respectively, on live PBMCs. D. Ratios of CD4^+^/CD8^+^ T cells. A complete summary of the immunophenotype is reported in Figure S2, extending those reported by Zuccato et al.^40^

Immunophenotyping of PBMCs was performed by flow cytometry analysing the following markers associated with the various lymphocyte subpopulations: CD3, CD4, CD8, CD14, CD19, CD25 and FOXP3. An example of the analysis of the immunophenotype is included in the (**Figure** **S1**). No major changes are detectable after Sirolimus treatment among monocytes (CD14^+^), B cells (CD19^+^) and the whole T‐cell compartment (CD3^+^). The new data and analyses shown in Figure 1 are in agreement with the results discussed by Zuccato et al.^40^ (see also **Figure** **S2**). Interestingly, CD4^+^ T cells, regulatory T cells (FOXP3^+^/CD25^+^) and activated CD8^+^ T cells (CD8^+^/CD25^+^) remain unchanged during therapeutic treatment with Sirolimus. Therefore, 6 months of chronic therapy with Sirolimus in Thalassemia patients did not perturb the percentages of different lymphoid and myeloid immune cells, with the exception of a slight and transient increase of CD8^+^ T cells after 3 months of therapy. Moreover, we did not observe permanent alterations at the level of the T‐cell compartment, but only a minor and transient rise in the frequency of CD8^+^ T cells and thus a subtle decrease of the CD4/CD8 ratio (Figure 1 and **Figure** **S2**). The results obtained confirmed and extended (by including the analysis of the Treg fraction) the data reported by Zuccato et al.,^40^ demonstrating that Sirolimus treatment does not induce negative alterations of the immunophenotype in β‐Thalassemia patients.

### 3.2 Effects of Sirolimus on naive and memory CD4+ and CD8+ T cells subpopulations

**FIGURE 2:**
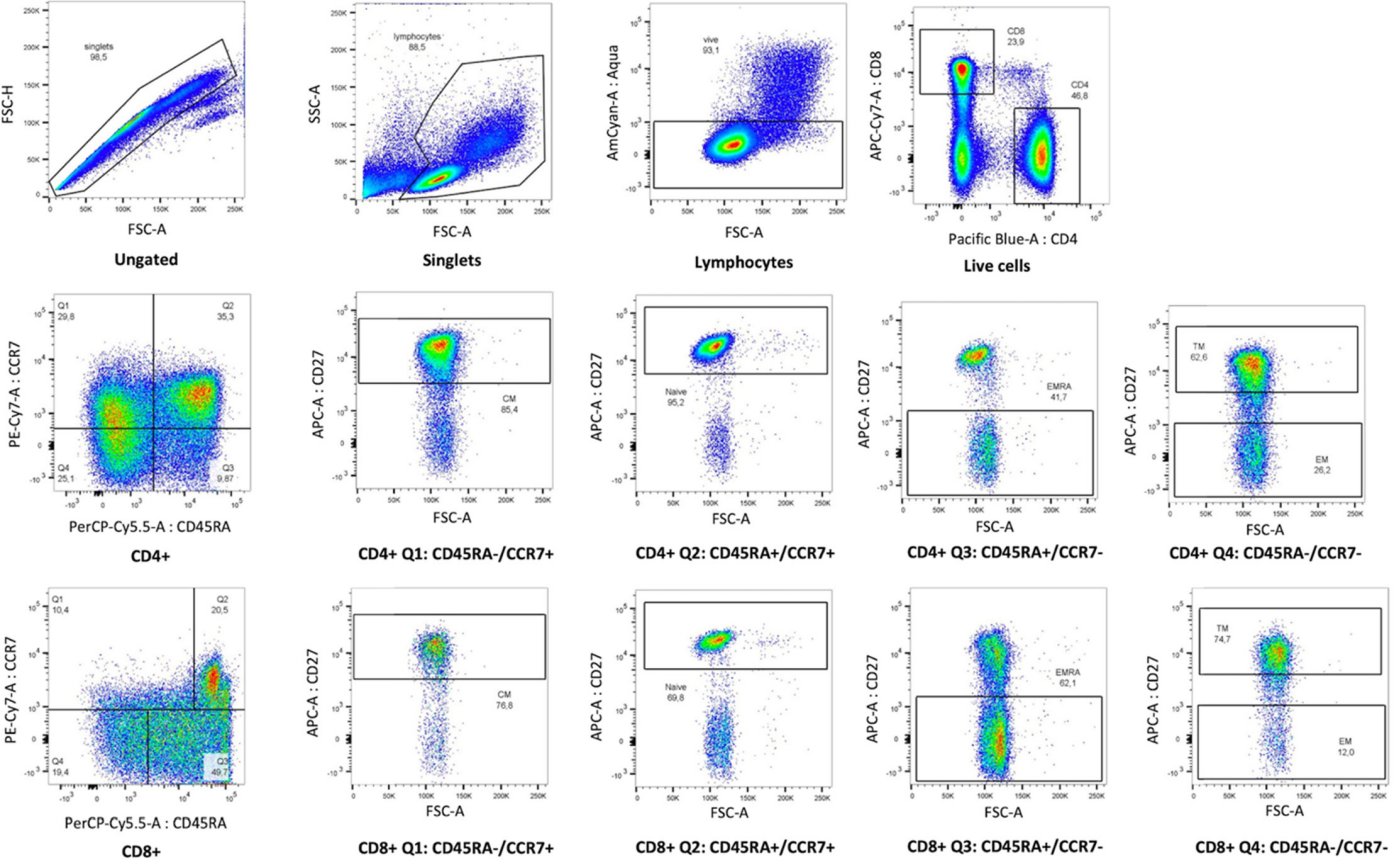
Representative data obtained from patient n.6 showing the employed gating strategy to detect memory T cells subpopulations (naïve, CM, TM, EM, EMRA). CM, central memory; EM, effector memory; EMRA, effector memory expressing CD45RA; TM, transitional memory

**FIGURE 3:**
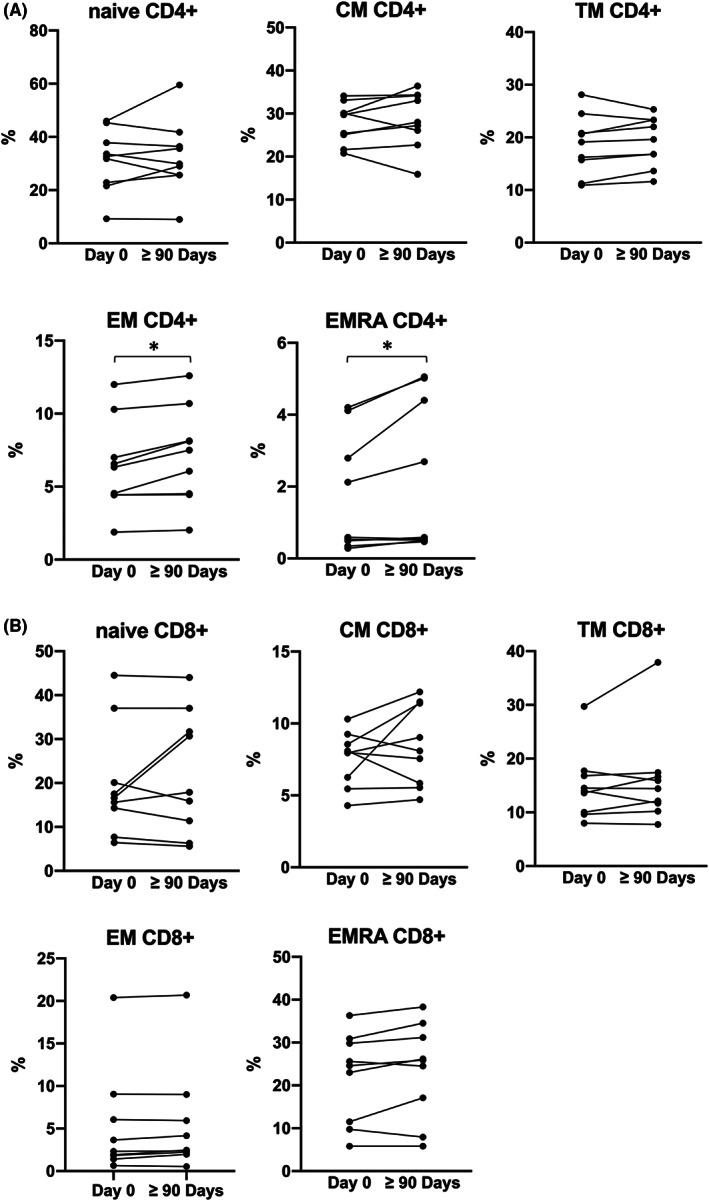
Summary representing the mean values of each memory T cells subset tested inside CD4+ (A) and CD8+ (B) subpopulation. (Day 0 *N* = 9, ≥90 Days *N* = 9). *p < .05, **p < .01. CM, central memory; EM, effector memory; EMRA, effector memory expressing CD45RA; TM, transitional memory

As depicted in the representative experiment shown in Figure 2, the analyses performed have been implemented for obtaining information on the following CD4^+^ (Figure 3A) and CD8^+^ (Figure 3B **)** subpopulations: naive, central memory (CM), transitional memory (TM) T cells, effector memory (EM) and effector memory expressing CD45RA (EMRA). These populations are of relevance, because identify T cells with different biological functions, and are clearly highlighted by antibodies staining. For instance, naive T cells express CD45RA receptor and co‐express CD27 and CCR7, central memory T cells (CM) co‐express CD27 and CCR7 but lack CD45RA. Effector memory T cells (EM) cells lack CD45RA, CD27 and CCR7 receptors, while EMRA T cells are terminally differentiated effector memory T‐cell re‐expressing CD45RA; transitional memory T cells (TM) are more differentiated than CM cells but not as fully differentiated as EM cells in terms of phenotype. Treatment of Sirolimus induced a slight but significant increase of both EM and EMRA CD4^+^ T cells (Figure 3A), while the subset distribution among CD8^+^ T cells remain unchanged (Figure 3B).

**FIGURE 4:**
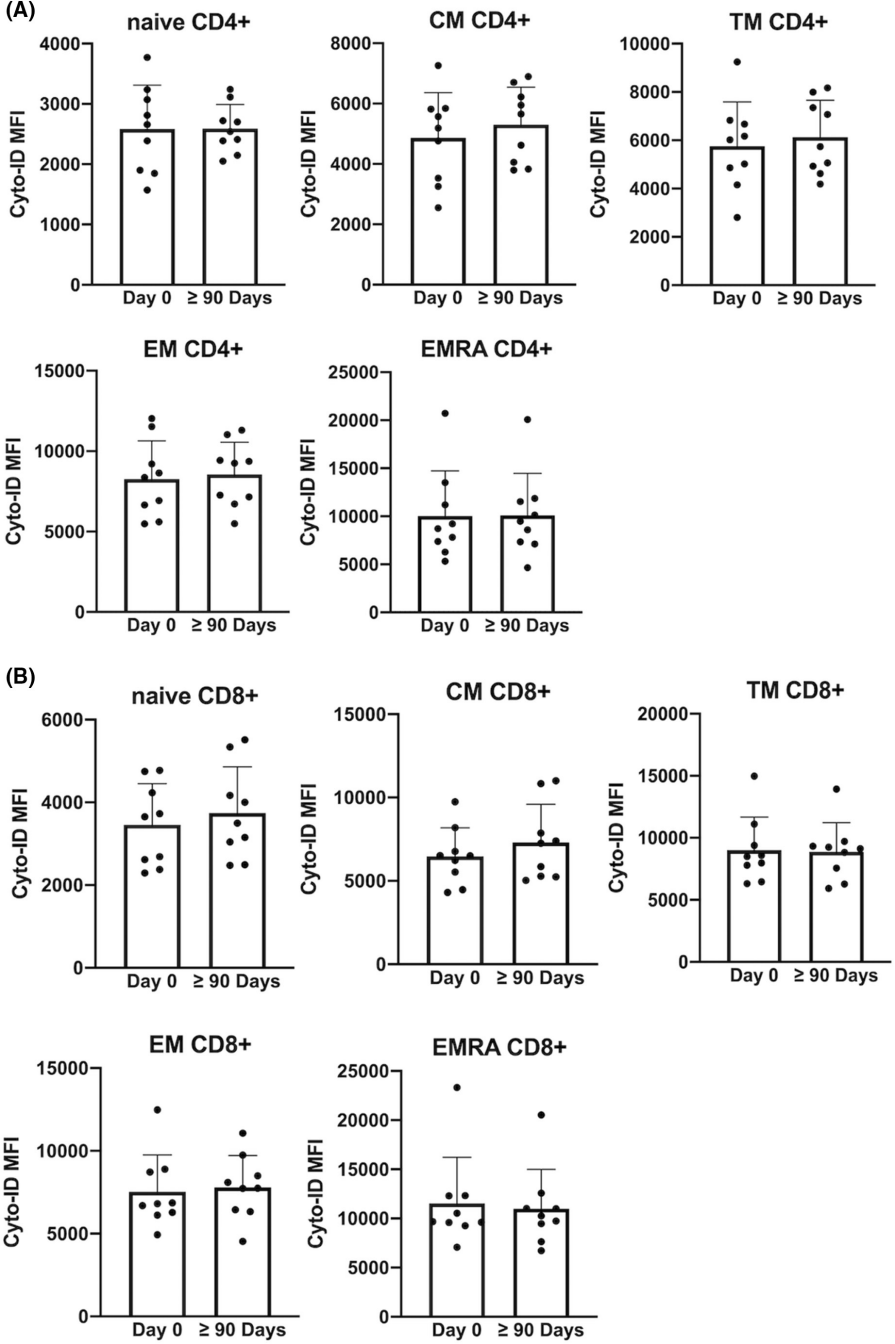
Autophagy levels of memory T cells subset tested. (Day 0 *N* = 9, ≥90 Days *N* = 9). *p < .05, **p < .01. CM, central memory; EM, effector memory; EMRA, effector memory expressing CD45RA; TM, transitional memory

Sirolimus, through mTOR inhibition, could increase autophagy levels,^54^, ^55^ and studies on humans and mice suggest that higher rates of autophagy sustain the long‐term survival of memory T cells.^10^, ^56^ For instance, experimental mice lacking the autophagy gene Atg7 in T cells failed to establish memory CD8^+^ T cells specific for influenza and MCMV infection.^56^ To investigate whether Sirolimus treatment affected autophagy levels in different T‐cell subsets, PBMCs isolated at Day 0 or after at least 90 days after treatment with Sirolimus were analysed for the contents of autophagic vesicles by flow cytometry. As shown in Figure 4, we observed a slight, although not nonsignificant, trend towards higher autophagy levels in both central memory CD4^+^ (Figure 4A) and CD8^+^ (Figure 4B) T cells, while autophagy levels of the other T‐cell subsets were unchanged.

### 3.3 Effects of Sirolimus treatment on activity of memory CD4+ T cells

**FIGURE 5:**
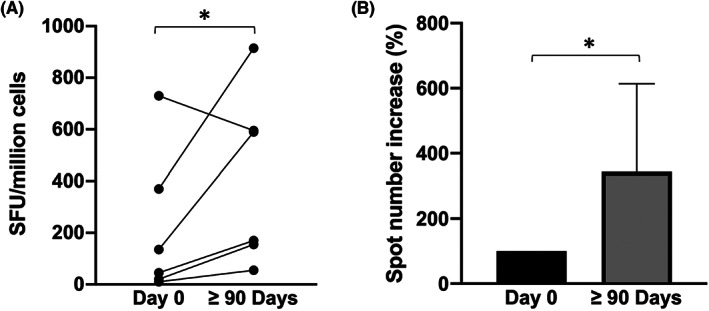
CD4^+^ memory T cells IFN‐γ release after stimulation with CEFT peptide in PBMCs isolated before to start Sirolimus administration and after three or more months of therapy. Data are presented as absolute values (A) or relatively to the starting point of each singular patient before drug administration (B). (Day 0 *N* = 6, ≥ 90 Days *N* = 6). *p < .05, **p < .01

The functionality of CD4^+^ T cells was analysed by the ELISpot technique following stimulation of the isolated PBMCs with the CEFT peptide pool. This peptide pool contains 24 MHC class II‐restricted viral peptides from human CMV, EBV, Influenza virus, Tetanus toxin and can selectively stimulate epitope‐specific human CD4^+^ T cells to produce IFN‐γ. The data obtained are summarized in Figure 5. In Figure 5A, the absolute values of epitope‐specific IFN‐γ secreting cells (SFU/million cells) are reported; in Figure 5B, the % increases in the spot numbers are indicated. The results demonstrate that after stimulation with CEFT peptides, epitope‐specific memory CD4^+^ T cells secerning IFN‐γ are increased after treatment with Sirolimus for 90 or more days in five patients on six.

### 3.4 Effects of Sirolimus on activity of memory CD8+ T cells of β‐Thalassemia patients

**FIGURE 6:**
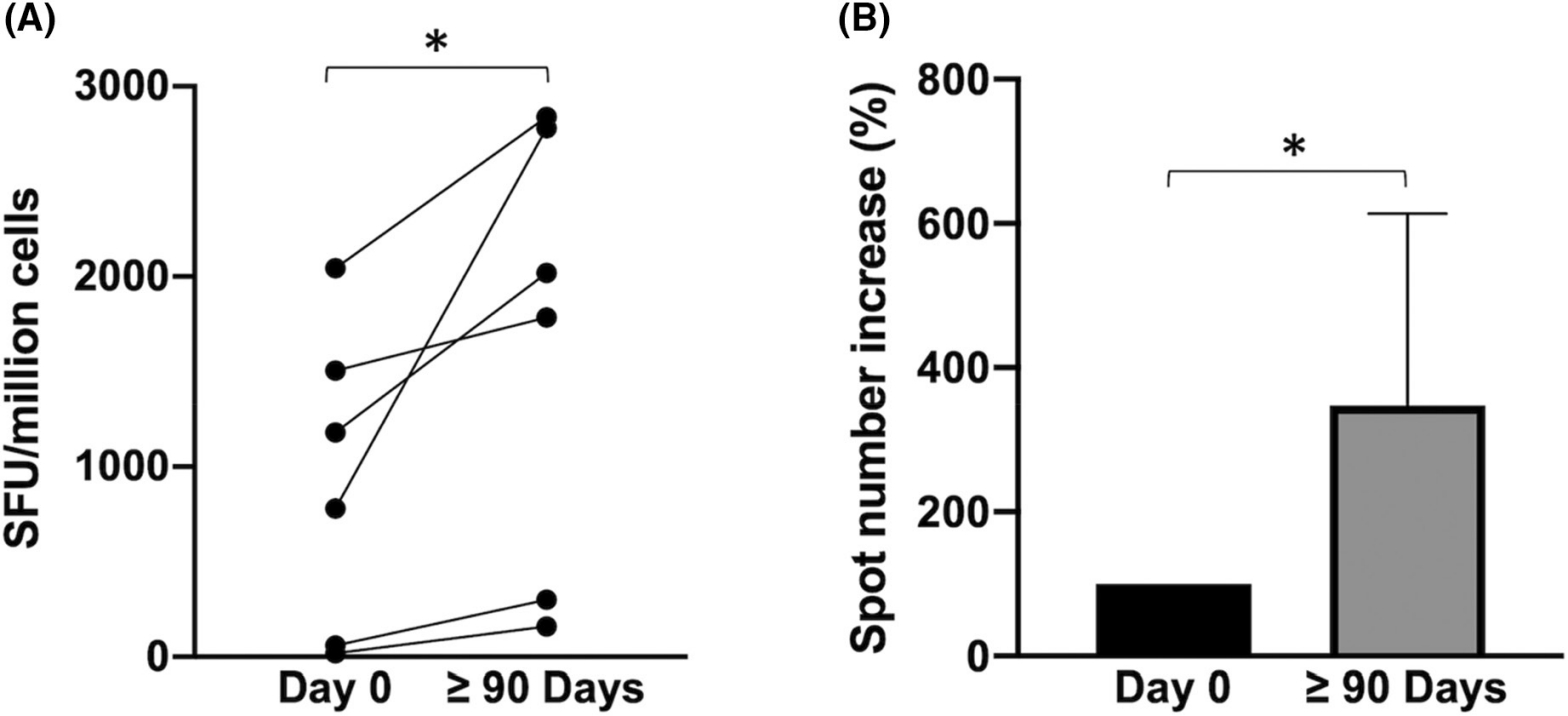
CD8^+^ memory T cells IFN‐γ release after stimulation with CEF peptide in PBMCs isolated before to start Sirolimus administration and after three or more months of therapy. Data are presented as absolute values (A) or relatively to the starting point of each singular patient before drug administration (B). (Day 0 *N* = 6, ≥90 Days *N* = 6). *p < .05, **p < .01

The activity of memory C8^+^ T cells was analysed following stimulation of the same isolated PBMCs employed for CD4^+^ memory T‐cell activity but stimulating them with the CEF peptide pool instead. The CEF peptide pool contains 32 MHC class I‐restricted viral peptides from human CMV, EBV and Influenza virus and can selectively stimulate epitope‐specific human CD8^+^ T cells to produce IFN‐γ. The data obtained are presented and summarized in Figure 6. The data obtained indicate that after three or more months of Sirolimus treatment, each patient showed a marked increase in the number of epitope‐specific memory CD8^+^ T cells releasing IFN‐γ following stimulation with CEF peptide pool (*p* = .031).

### 3.5 Effects of Sirolimus on the plasmatic secretome profile of treated β‐Thalassemia patients

**FIGURE 7:**
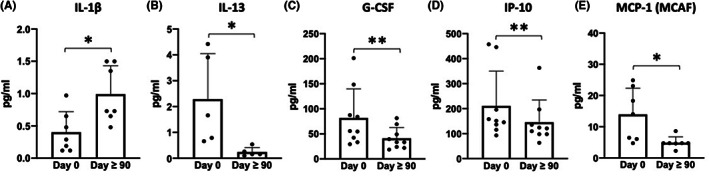
Content of IL‐1β (A), IL‐13 (B), G‐CSF (C), IP‐10 (D) and MCP‐1(MCAF) (E) in plasma samples isolated at Day 0 and after 90 or more days from the beginning of sirolimus intake by treated patients. (A) *N* = 7; (B) *N* = 5; (C and D) *N* = 9; (E) *N* = 7. The Bioplex method and the complete set of the analysed proteins related to this Figure can be found in the Supplementary Materials (SM1 and Figure S3, respectively). *p < .05, **p < .01

In order to better characterize potential systemic effects of Sirolimus treatment on the immune compartment, components of the secretome present in the plasma samples of Sirolimus‐treated patients were analysed by Bioplex technology, that allows the parallel analysis of 27 secreted proteins (interleukins, chemokines, growth factors).^53^ In fact, it is well‐known that cytokines and their signalling pathways exert potent effects on T‐cell metabolism, activation and differentiation.^57^ In particular, several Th1, Th2, pro‐inflammatory and anti‐inflammatory cytokines were measured. The complete set of data obtained are included in **Figure** **S3**, while Figure 7 reports the plasma content of the protein exhibiting the most significant differences comparing plasma collected before and at least 90 days after treatment (IL‐1β, IL‐13, G‐CSF, IP‐10) and MCP1(MCAF). A significant decrease of plasma content (pg/ml) of Monocyte Chemoattractant Protein‐1 (MCP‐1), of the Th2 cytokine IL‐13 and of Granulocyte colony‐stimulating factor (G‐CSF) was observed after Sirolimus treatment. Interestingly, G‐CSF is a strong immune regulator that inhibits CD8^+^ responses and promotes Th2 differentiation.^58^ Moreover, patients displayed, after Sirolimus treatment, significant lower levels of the pro‐inflammatory chemokine interferon gamma‐induced protein 10 (IP‐10) but increased amounts of IL‐1β. Importantly, IL‐1β was the only pro‐inflammatory cytokine whose levels were augmented by Sirolimus (see for instance the unaltered levels of MIP‐1α or TNF‐α, reported in **Figure** **S3**), suggesting a reversion of immunosuppression rather than the induction of a low‐grade inflammatory state.

Together, these data suggest that Sirolimus could systemically restore the cytokine milieu of β‐Thalassemia patients dampening Th2 responses and immune suppression environments without provoking excessive inflammation levels.

## 4 DISCUSSION

The main results of the present study are that treatment of β‐Thalassemia patients with Sirolimus has a positive impact on the biological activity of memory CD4^+^ and CD8^+^ T cells. This conclusion is outlined by the data depicted in **Figures** **5** and **6**, that indicate increased number of epitope‐specific CD4^+^ and of CD8^+^ memory T cells releasing IFN‐γ following stimulation with antigenic stimuli present in immunological memory. These data are to our knowledge novel and of great interest in consideration of the fact that β‐Thalassemia patients are considered prone to immune deficiency.^47^

Immune dysfunctions characterizing thalassemia patients include changes in lymphocyte subsets, such as the accumulation of suppressor T cells and the reduced proliferative capacity and numbers of T helper cells, as well as the defective activity of natural killer (NK) cells. Similarly, an altered humoral immunity has been shown in patients with β‐Thalassemia.^48^ Accordingly, β‐Thalassemia‐associated immunosuppression should be actively targeted to protect these patients.

In this context, our results are in line with several observations reporting that mTOR inhibitors (such as Sirolimus) are Immunomodulatory molecules of great interest in the context of vaccination, since they might have an effect on the cellular memory response, possibly enhancing long‐term effects of vaccines. Accordingly, Sirolimus were found by Mannick et al. to improve responses to influenza vaccination in adults, with benefits possibly persisting for a year after treatment.^59^, ^60^

This is an important issue of the vaccination campaign against the severe acute respiratory syndrome coronavirus (SARS‐CoV‐2), causing the dramatic COVID‐19 (coronavirus disease 2019) pandemic.^61^ In this respect, one of the great concerns about COVID‐19 vaccination is the length of time this approach will protect the vaccinated population from infection by SARS‐CoV‐2 and from the development of severe COVID‐19 associated symptoms. In the context of the possible effects of immunomodulatory molecules on the effectiveness of vaccines, mTOR inhibitors (such as Everolimus and Sirolimus) are of great interest, and a possible use of Sirolimus to improve response to SARS‐CoV‐2 vaccination has been proposed by our group.^62^

In this context, Netti et al.^63^ published a very interesting observation demonstrating that mTOR inhibitors were able to increase SARS‐CoV‐2‐specific T cell‐derived IFN‐γ release in kidney transplant recipient (KTR) taking the mRNA BNT162b2 (Pfizer‐BioNTech) vaccine. The conclusion of this study suggested that the presence of mTOR inhibitors is associated with a better immune response to COVID‐19 vaccine in transplanted patient compared to therapy lacking mTOR inhibitors. These findings are consistent with a potential beneficial role of mTOR inhibitors as modulators of immune response to COVID‐19 vaccine.

Our present study confirms the possible beneficial role of the mTOR inhibitor Sirolimus on the biological activity of memory T cells in β‐Thalassemia patients. The results obtained demonstrate that Sirolimus preserves and enhances the number and activity of epitope‐specific CD4^+^ and CD8^+^ T cells without depleting the naive subset. Other possible positive effects observed during Sirolimus treatment included CD8^+^ T cells and EM and EMRA CD4^+^ T cells percentage increase respect to before starting therapy.

Although the levels of autophagy measured in CD4 and CD8 memory subpopulations tended to rise, it remains unclear if Sirolimus dosage adopted in this study is sufficient to promote autophagy in T cells and if this is directly related to the biological effect of enhancing memory T‐cell releasing IFN‐γ following stimulation; future experiments might consider using Chloroquine or Bafilomycin in order to better detect autophagic flux,^64^ and future clinical trials might consider increasing Sirolimus dosage to determine whether higher levels of autophagy are obtained in memory T cells.

Our data do not clarify mitochondrial overall activity, metabolism and possible activation of mitophagy in CD4^+^ and CD8^+^ memory T‐cell subpopulations from Sirolimus‐treated patients, as reported in elsewhere published studies.^65^, ^66^, ^67^ However, the results obtained by Bioplex analysis of cytokines, chemokines and growth factors demonstrated that Sirolimus treatment leads to significant changes in the content of IL‐1β, G‐CSF, IL‐13 and IP‐10, all involved in regulating T‐cell metabolism and function^57^, ^68^, ^69^, ^70^ (Figure 7 and **Figure** **S3**). The increase of IL‐1β (Figure 7A, *p* = .028) is of interest, considering the fact that IL‐1β is required for antigen‐specific T‐cell activation. In fact, analysis of in vivo gene expression in CD4^+^ T cells stimulated with IL‐1β revealed that IL‐1β caused gene expression changes consistent with the up‐regulation of pathways involved in cell replication, cell survival and enhanced energy metabolism.^71^ Interestingly, effector memory CD4^+^ T cells are among T‐cell subsets more susceptible to the effects of IL‐1β,^72^ which includes increased peripheral survival and recall memory responses. This would be consistent with the increased levels of EM CD4^+^ T cells and augmented CD4 responses to memory antigens after Sirolimus treatment. Moreover, IL‐1β is crucial for CD8^+^ T‐cell activation and antigen‐specific responses.^73^

Of interest is also the decrease of IL‐13 (Figure 7B, *p* = .043) and IP‐10 (Figure 7D, *p* = .008) in Sirolimus‐treated patients. Concerning IL‐13, it has been reported that blocking IL‐13 enhances the therapeutic effect of antigen‐specific immunotherapy by enforcing the activity of CD41 T cells.^69^ Concerning IP‐10, it is established that high levels of this protein decrease T‐cell function.^70^ The inhibitory effects of Sirolimus on IL13 and IP‐10 sustain the concept that this mTOR inhibitor might divert helper cells towards Th1, rather than Th2, polarization and enhance T‐cell function, as indicated by the experiments reported in **Figures** **5** and **6**.

Finally, a remarkable decrease of G‐CSF was observed (Figure 7C, *p* = .008). Interestingly, G‐CSF has been demonstrated to be a strong immune regulator of T cells, being C‐CSF treatment able to potently reduce reactivity of T cells and imbalance the Th1/Th2 ratio.^58^ This exploratory experimental plan should stimulate further studies on the effects of Sirolimus on immunomodulation, especially considering the pro‐inflammatory status of β‐Thalassemia patients.^74^

Our data do not clarify whether the effect of Sirolimus was mediated directly on memory T cells or indirectly on APC. This cannot be in theory excluded, as it is known that autophagy processes regulate peptide presentation in professional antigen‐presenting cells, which mediate thymocyte selection.^67^ Nonetheless, our secretome data tend to suggest that Sirolimus can impact on the cytokine milieu, and this translates in increased levels of EM CD4^+^ T cells and of Th1 CD4 and CD8 recall responses.

In any case, and whatever being the mechanism of action, the beneficial effect of Sirolimus treatment on COVID‐19 vaccination might be of great interest for managing β‐Thalassemia, as an increased mortality risk from COVID‐19 was observed in Italian patients affected by hemoglobinopathies, including β‐Thalassemia.^75^ A further issue in β‐Thalassemia is premature ageing of the immune system, that has been suggested to affect the response to SARS‐CoV‐2 mRNA vaccine.^76^ Considering that autophagy and mitophagy in T cells decrease with age,^10^ the induction/potentiation of these biological processes in elderly should be considered of great importance.

It must be noted that in addition to possible boosting effects on vaccination efficacy/duration, mTOR inhibition is under investigation also as a new therapeutic option in the management of SARS‐CoV‐2 infection.^77^ The study of the possible effects of Sirolimus on immune response to COVID‐19 vaccine is at present ongoing within the clinical trial NCT04247750 (Treatment of β‐Thalassemia Patients with Rapamycin: From Pre‐clinical Research to a Clinical Trial), that has recruited patients vaccinated against SARS‐CoV‐2 using the mRNA BNT162b2 (Pfizer‐BioNTech) or mRNA‐1273 (Moderna) vaccines and started Sirolimus treatment after completing the vaccination procedure. The impact of our study on the management of β‐Thalassemia patients is relevant for the following considerations: (a) an increased susceptibility to infections (including SARS‐CoV‐2 infection) has been described in unvaccinated β‐thalassemia heterozygous subjects, highlighting the importance of vaccination and boosting the protective effects of vaccines in TM patients^43^; (b) sirolimus has been suggested to ameliorate haematological parameters in treated β‐thalassemia subjects, including the decreases of the excess of free α‐globin chains, possibly caused by activation of autophagy.^40^, ^78^

## AUTHOR CONTRIBUTIONS

**Matteo Zurlo:** Conceptualization (lead); data curation (lead); formal analysis (lead); investigation (supporting); methodology (lead); writing – original draft (supporting); writing – review and editing (supporting). **Francesco Nicoli:** Conceptualization (lead); data curation (lead); formal analysis (lead); supervision (lead); writing – original draft (supporting); writing – review and editing (supporting). **Davide Proietto:** Data curation (supporting); formal analysis (supporting); methodology (supporting); writing – review and editing (supporting). **Beatrice Dallan:** Data curation (supporting); formal analysis (supporting); investigation (supporting); methodology (supporting); writing – review and editing (supporting). **Cristina Zuccato:** Data curation (supporting); formal analysis (supporting); investigation (supporting); methodology (supporting); validation (supporting); writing – review and editing (supporting). **Lucia Carmela Cosenza:** Data curation (supporting); formal analysis (supporting); investigation (supporting); methodology (supporting); validation (supporting); writing – review and editing (supporting). **Jessica Gasparello:** Data curation (supporting); formal analysis (supporting); investigation (supporting); methodology (supporting); validation (supporting); writing – review and editing (supporting). **Chiara Papi:** Data curation (supporting); formal analysis (supporting); investigation (supporting); methodology (supporting); writing – review and editing (supporting). **Monica Borgatti:** Conceptualization (supporting); data curation (supporting); formal analysis (supporting); supervision (lead); writing – review and editing (supporting). **Alessia Finotti:** Conceptualization (supporting); data curation (supporting); formal analysis (supporting); funding acquisition (lead); supervision (lead); writing – review and editing (supporting). **Roberto Gambari:** Conceptualization (lead); data curation (supporting); formal analysis (lead); funding acquisition (lead); project administration (lead); resources (lead); supervision (lead); writing – original draft (lead); writing – review and editing (lead). **Chiara Scapoli:** Formal analysis (equal). **Elisabetta d'Aversa:** Formal analysis (equal).

## FUNDING INFORMATION

Supported by Wellcome Trust (United Kingdom, Innovator Award 208872/Z/17/Z), by AIFA (Agenzia Italiana del Farmaco, Italy, AIFA‐2016‐02364887) and by MUR‐FISR COVID‐miRNAPNA Project (FISR2020IP_04128).

## CONFLICT OF INTEREST

The authors declare that they have no conflict of interest.

## PATIENT CONSENT FOR PUBLICATION

All patients signed an informed consent, that includes their consent to publication.

## Supporting information


Data S1

## Data Availability

The data that support the findings of this study are available from the corresponding author upon reasonable request.

## References

[1] Keating R , Hertz T , Wehenkel M , et al. The kinase mTOR modulates the antibody response to provide cross‐protective immunity to lethal infection with influenza virus. Nat Immunol. 2013;14(12):1266‐1276. 24141387 10.1038/ni.2741 PMC3883080

[2] Cohen J . Infectious disease. Immune suppressant unexpectedly boosts flu vaccine. Science. 2013;342(6157):413. 24159023 10.1126/science.342.6157.413

[3] Frasca D , Blomberg BB . Inflammaging decreases adaptive and innate immune responses in mice and humans. Biogerontology. 2016;17(1):7‐19. 25921609 10.1007/s10522-015-9578-8 PMC4626429

[4] Blasi F , Gramegna A , Sotgiu G , et al. SARS‐CoV‐2 vaccines: a critical perspective through efficacy data and barriers to herd immunity. Respir Med. 2021;180:106355. 33721697 10.1016/j.rmed.2021.106355 PMC7935673

[5] Cunningham AL , McIntyre P , Subbarao K , et al. Vaccines for older adults. BMJ. 2021;372:n188. 33619170 10.1136/bmj.n188

[6] Bak S , Tischer S , Dragon A , et al. Selective effects of mTOR inhibitor Sirolimus on Naïve and CMV‐specific T cells extending its applicable range beyond immunosuppression. Front Immunol. 2018;9:2953. 30619313 10.3389/fimmu.2018.02953 PMC6304429

[7] Jung JW , Veitch M , Bridge JA , et al. Clinically‐relevant rapamycin treatment regimens enhance CD8+ effector memory T cell function In the skin and allow their infiltration into cutaneous squamous cell carcinoma. Onco Targets Ther. 2018;7(9):e1479627. 10.1080/2162402X.2018.1479627 PMC6140608 30228949

[8] Amiel E , Everts B , Freitas TC , et al. Inhibition of mechanistic target of rapamycin promotes dendritic cell activation and enhances therapeutic autologous vaccination in mice. J Immunol. 2012;189(5):2151‐2158. 22826320 10.4049/jimmunol.1103741 PMC3424310

[9] Araki K , Youngblood B , Ahmed R . The role of mTOR in memory CD8 T‐cell differentiation. Immunol Rev. 2010;235(1):234‐243. 20536567 10.1111/j.0105-2896.2010.00898.x PMC3760155

[10] Macian F . Autophagy in T cell function and aging. Front Cell Dev Biol. 2019;7:213. 31632966 10.3389/fcell.2019.00213 PMC6783498

[11] Kahan BD . Sirolimus: a comprehensive review. Expert Opin Pharmacother. 2001;2(11):1903‐1917. 11825325 10.1517/14656566.2.11.1903

[12] Sehgal SN . Sirolimus: its discovery, biological properties, and mechanism of action. Transplant Proc. 2003;35(3 Suppl):7 S‐14 S. 10.1016/s0041-1345(03)00211-2 12742462

[13] Vasquez EM . Sirolimus: a new agent for prevention of renal allograft rejection. Am J Health Syst Pharm. 2000;57(5):437‐451. 10711524 10.1093/ajhp/57.5.437

[14] Hernández D , Martínez D , Gutiérrez E , et al. Clinical evidence on the use of anti‐mTOR drugs in renal transplantation. Nefrologia. 2011;31:27‐34. 21270910 10.3265/Nefrologia.pre2010.Jul.10512

[15] Schaffer SA , Ross HJ . Everolimus: efficacy and safety in cardiac transplantation. Expert Opin Drug Saf. 2010;9(5):843‐854. 20701555 10.1517/14740338.2010.511611

[16] Tang CY , Shen A , Wei XF , et al. Everolimus in de novo liver transplant recipients: a systematic review. Hepatobiliary Pancreat Dis Int. 2015;14(5):461‐469. 26459721 10.1016/s1499-3872(15)60419-2

[17] Ji L , Xie W , Zhang Z . Efficacy and safety of sirolimus in patients with systemic lupus erythematosus: a systematic review and meta‐analysis. Semin Arthritis Rheum. 2020;50(5):1073‐1080. 32911286 10.1016/j.semarthrit.2020.07.006

[18] Wang Q , Luo M , Xiang B , Chen S , Ji Y . The efficacy and safety of pharmacological treatments for lymphangioleiomyomatosis. Respir Res. 2020;21(1):55. 32059669 10.1186/s12931-020-1316-3 PMC7023761

[19] Sasongko TH , Ismail NF , Zabidi‐Hussin Z . Rapamycin and rapalogs for tuberous sclerosis complex. Cochrane Database Syst Rev. 2016;7(7):CD011272. 27409709 10.1002/14651858.CD011272.pub2 PMC6458010

[20] Graillon T , Sanson M , Campello C , et al. Everolimus and octreotide for patients with recurrent meningioma: results from the phase II CEVOREM trial. Clin Cancer Res. 2020;26(3):552‐557. 31969329 10.1158/1078-0432.CCR-19-2109

[21] Gallo M , Malandrino P , Fanciulli G , et al. Everolimus as first line therapy for pancreatic neuroendocrine tumours: current knowledge and future perspectives. J Cancer Res Clin Oncol. 2017;143(7):1209‐1224. 28405826 10.1007/s00432-017-2407-5 PMC11819053

[22] Manohar PM , Beesley LJ , Taylor JM , et al. Retrospective study of Sirolimus and cyclophosphamide in patients with advanced differentiated thyroid cancers. J Thyroid Disord Ther. 2015;4(3):188. 27088062 10.4172/2167-7948.1000188 PMC4831630

[23] Hortobagyi GN . Everolimus plus exemestane for the treatment of advanced breast cancer: a review of subanalyses from BOLERO‐2. Neoplasia. 2015;17(3):279‐288. 25810012 10.1016/j.neo.2015.01.005 PMC4372651

[24] Merli M , Ferrario A , Maffioli M , Arcaini L , Passamonti F . Everolimus in diffuse large B‐cell lymphomas. Future Oncol. 2015;11(3):373‐383. 25675120 10.2217/fon.14.264

[25] Motzer RJ , Escudier B , Oudard S , et al. Phase 3 trial of everolimus for metastatic renal cell carcinoma: final results and analysis of prognostic factors. Cancer. 2010;116(18):4256‐4265. 20549832 10.1002/cncr.25219

[26] Mischiati C , Sereni A , Lampronti I , et al. Rapamycin‐mediated induction of gamma‐globin mRNA accumulation in human erythroid cells. Br J Haematol. 2004;126(4):612‐621. 15287957 10.1111/j.1365-2141.2004.05083.x

[27] Fibach E , Bianchi N , Borgatti M , et al. Effects of rapamycin on accumulation of alpha‐, beta‐ and gamma‐globin mRNAs in erythroid precursor cells from beta‐thalassaemia patients. Eur J Haematol. 2006;77(5):437‐441. 16939628 10.1111/j.1600-0609.2006.00731.x

[28] Zuccato C , Bianchi N , Borgatti M , et al. Everolimus is a potent inducer of erythroid differentiation and gamma‐globin gene expression in human erythroid cells. Acta Haematol. 2007;117(3):168‐176. 17148936 10.1159/000097465

[29] Pecoraro A , Troia A , Calzolari R , et al. Efficacy of rapamycin as inducer of Hb F in primary erythroid cultures from sickle cell disease and β‐thalassemia patients. Hemoglobin. 2015;39(4):225‐229. 26016899 10.3109/03630269.2015.1036882

[30] Gaudre N , Cougoul P , Bartolucci P , et al. Improved fetal hemoglobin with mTOR inhibitor‐based immunosuppression in a kidney transplant recipient with sickle cell disease. Am J Transplant. 2017;17(8):2212‐2214. 28276629 10.1111/ajt.14263

[31] Al‐Khatti AA , Alkhunaizi AM . Additive effect of sirolimus and hydroxycarbamide on fetal haemoglobin level in kidney transplant patients with sickle cell disease. Br J Haematol. 2019;185(5):959‐961. 30407620 10.1111/bjh.15665

[32] Thein SL . The molecular basis of β‐thalassemia. Cold Spring Harb Perspect Med. 2013;3(5):a011700. 23637309 10.1101/cshperspect.a011700 PMC3633182

[33] Nienhuis AW , Nathan DG . Pathophysiology and clinical manifestations of the β‐Thalassemias. Cold Spring Harb Perspect Med. 2012;2(12):a011726. 23209183 10.1101/cshperspect.a011726 PMC3543079

[34] Imamura T . The molecular basis of the thalassemia syndromes. Jpn J Hum Genet. 1977;22:113‐128. 10.1007/BF01874278 604562

[35] Sharma DC , Singhal S , Woike P , Rai S , Yadav M , Gaur R . Hereditary persistence of fetal hemoglobin. Asian J Transfus Sci. 2020;14(2):185‐186. 33767547 10.4103/ajts.AJTS_71_16 PMC7983139

[36] Bethlenfalvay NC , Motulsky AG , Ringelhann B , Lehmann H , Humbert JR , Konotey‐Ahulu FI . Hereditary persistence of fetal hemoglobin, beta thalassemia, and the hemoglobin delta‐beta locus: further family data and genetic interpretations. Am J Hum Genet. 1975;27(2):140‐154. 1124762 PMC1762752

[37] Demirci S , Leonard A , Tisdale JF . Genome editing strategies for fetal hemoglobin induction in beta‐hemoglobinopathies. Hum Mol Genet. 2020;29(R1):R100‐R106. 32406490 10.1093/hmg/ddaa088 PMC7673473

[38] Perrine SP , Pace BS , Faller DV . Targeted fetal hemoglobin induction for treatment of beta hemoglobinopathies. Hematol Oncol Clin North Am. 2014;28(2):233‐248. 24589264 10.1016/j.hoc.2013.11.009

[39] Gamberini MR , Prosdocimi M , Gambari R . Sirolimus for treatment of β‐thalassemia: from pre‐clinical studies to the design of clinical trials. Health Educ Pub Health. 2021;4:425‐435.

[40] Zuccato C , Cosenza LC , Zurlo M , et al. Expression of γ‐globin genes in β‐thalassemia patients treated with sirolimus: results from a pilot clinical trial (Sirthalaclin). Ther Adv Hematol. 2022;13:204062072211006. 10.1177/20406207221100648 PMC9218916 35755297

[41] Zafari M , Rad MTS , Mohseni F , Nikbakht N . β‐Thalassemia major and Coronavirus‐19, mortality and morbidity: a systematic review study. Hemoglobin. 2021;45(1):1‐4. 33317358 10.1080/03630269.2020.1857266

[42] Soltani S , Zakeri A , Tabibzadeh A , et al. A literature review on the parvovirus B19 infection in sickle cell anemia and β‐thalassemia patients. Trop Med Health. 2020;48(1):96. doi:10.1186/s41182-020-00284-x 33292852 PMC7709306

[43] Sotiriou S , Samara AA , Vamvakopoulou D , et al. Susceptibility of β‐thalassemia heterozygotes to COVID‐19. J Clin Med. 2021;10(16):3645. 34441941 10.3390/jcm10163645 PMC8397014

[44] Kandi V , Vinjamuri SR , Tanikella BP . Hepatitis C viral infection among Beta‐thalassemia patients: a study from the Centre for Excellence in thalassemia and other blood disorders. Cureus. 2021;13(7):e16207. 34367809 10.7759/cureus.16207 PMC8340578

[45] Jahromi AS , Rahmanian K . Immunity to tetanus in major beta thalassemia patients. Clin Exp Vaccine Res. 2015;4(2):184‐188. 26273577 10.7774/cevr.2015.4.2.184 PMC4524903

[46] Jahromi AS , Rahmanian K , Davami MH , Zabetian H , Yousefi A , Madani A . Natural immunity against Haemophilus influenza type B in splenectomised Beta‐thalassaemia children. Pak J Biol Sci. 2014;17(11):1190‐1194. 26027165 10.3923/pjbs.2014.1190.1194

[47] Farmakis D , Giakoumis A , Polymeropoulos E , Aessopos A . Pathogenetic aspects of immune deficiency associated with beta‐thalassemia. Med Sci Monit. 2003;9(1):RA19‐RA22. 12552254

[48] Ghaffari J , Abediankenari S , Nasehi M . Thalassemia and immune system dysfunction‐review article. Int J Curr Res. 2011;3:105‐108.

[49] Ezer U , Gulderen F , Culha VK , Akgul N , Gurbuz O . Immunological status of thalassemia syndrome. Pediatr Hematol Oncol. 2002;19:51‐58. 11787867 10.1080/088800102753356194

[50] Cappellini MD , Cohen A , Porter J , Taher A , Viprakasit V , eds. Guidelines for the Management of Transfusion Dependent Thalassaemia (TDT). 3rd ed. Nicosia (CY); 2014. 25610943

[51] Nicoli F , Papagno L , Frere JJ , et al. Naïve CD8^+^ T‐cells engage a versatile metabolic program upon activation in humans and differ energetically from memory CD8^+^ T‐cells. Front Immunol. 2018;9:2736. 30619240 10.3389/fimmu.2018.02736 PMC6308131

[52] Gallerani E , Proietto D , Dallan B , et al. Impaired priming of SARS‐CoV‐2‐specific Naïve CD8+ T cells in older subjects. Front Immunol. 2021;12:693054. 34326844 10.3389/fimmu.2021.693054 PMC8315546

[53] Gasparello J , D'Aversa E , Papi C , et al. Sulforaphane inhibits the expression of interleukin‐6 and interleukin‐8 induced in bronchial epithelial IB3‐1 cells by exposure to the SARS‐CoV‐2 spike protein. Phytomedicine. 2021;87:153583. 34033999 10.1016/j.phymed.2021.153583 PMC8095027

[54] Wang H , Fu J , Xu X , Yang Z , Zhang T . Rapamycin activates Mitophagy and alleviates cognitive and synaptic plasticity deficits in a mouse model of Alzheimer's disease. J Gerontol A Biol Sci Med Sci. 2021;76:1707‐1713. 34003967 10.1093/gerona/glab142

[55] Huang C , Zhang Y , Kelly DJ , et al. Thioredoxin interacting protein (TXNIP) regulates tubular autophagy and mitophagy in diabetic nephropathy through the mTOR signaling pathway. Sci Rep. 2016;6:29196. 27381856 10.1038/srep29196 PMC4933928

[56] Puleston DJ , Zhang H , Powell TJ , et al. Autophagy is a critical regulator of memory CD8(+) T cell formation. Elife. 2014;3:e03706. 25385531 10.7554/eLife.03706 PMC4225493

[57] Nambu A , Nakae S , Iwakura Y . IL‐1beta, but not IL‐1alpha, is required for antigen‐specific T cell activation and the induction of local inflammation in the delayed‐type hypersensitivity responses. Int Immunol. 2006;18(5):701‐712. doi:10.1093/intimm/dxl007 Epub March 28, 2006. 16569679

[58] Franzke A , Piao W , Lauber J , et al. G‐CSF as immune regulator in T cells expressing the G‐CSF receptor: implications for transplantation and autoimmune diseases. Blood. 2003;102(2):734‐739. 12676791 10.1182/blood-2002-04-1200

[59] Mannick JB , Del Giudice G , Lattanzi M , et al. mTOR inhibition improves immune function in the elderly. Sci Transl Med. 2014;6(268):268ra179. 10.1126/scitranslmed.3009892 25540326

[60] Mannick JB , Morris M , Hockey HP , et al. TORC1 inhibition enhances immune function and reduces infections in the elderly. Sci Transl Med. 2018;10(449):eaaq1564. 29997249 10.1126/scitranslmed.aaq1564

[61] Huang C , Wang Y , Li X , et al. Clinical features of patients infected with 2019 novel coronavirus in Wuhan, China. Lancet, [published correction appears in lancet. January 30, 2020]. 2020;395(10223):497‐506. 31986264 10.1016/S0140-6736(20)30183-5 PMC7159299

[62] Zurlo M , Nicoli F , Borgatti M , Finotti A , Gambari R . Possible effects of sirolimus treatment on the long‐term efficacy of COVID‐19 vaccination in patients with β‐thalassemia: a theoretical perspective. Int J Mol Med. 2022;49:33. 35059731 10.3892/ijmm.2022.5088

[63] Netti GS , Infante B , Troise D , et al. mTOR inhibitors improve both humoral and cellular response to SARS‐CoV‐2 messenger RNA BNT16b2 vaccine in kidney transplant recipients. Am J Transplant. 2022;22:1475‐1482. doi:10.1111/ajt.16958 35038362 PMC9303518

[64] Gottlieb RA , Andres AM , Sin J , Taylor DP . Untangling autophagy measurements: all fluxed up. Circ Res. 2015;116(3):504‐514. 25634973 10.1161/CIRCRESAHA.116.303787 PMC4313387

[65] Wei Y , Zhang YJ , Cai Y , Xu MH . The role of mitochondria in mTOR‐regulated longevity. Biol Rev Camb Philos Soc. 2015;90:167‐181. 24673778 10.1111/brv.12103

[66] Buck MD , O'Sullivan D , Klein Geltink RI , et al. Mitochondrial dynamics controls T cell fate through metabolic programming. Cell. 2016;166(1):63‐76. 27293185 10.1016/j.cell.2016.05.035 PMC4974356

[67] Bronietzki AW , Schuster M , Schmitz I . Autophagy in T‐cell development, activation and differentiation. Immunol Cell Biol. 2015;93(1):25‐34. 25287445 10.1038/icb.2014.81

[68] Bunse CE , Borchers S , Varanasi PR , et al. Impaired functionality of antiviral T cells in G‐CSF mobilized stem cell donors: implications for the selection of CTL donor. PLoS One. 2013;8(12):e77925. 24324576 10.1371/journal.pone.0077925 PMC3850912

[69] Yang L , Xu LZ , Liu ZQ , et al. Interleukin‐13 interferes with activation‐induced t‐cell apoptosis by repressing p53 expression. Cell Mol Immunol. 2016;13(5):669‐677. 26189367 10.1038/cmi.2015.50 PMC5037282

[70] Ramirez LA , Arango TA , Thompson E , Naji M , Tebas P , Boyer JD . High IP‐10 levels decrease T cell function in HIV‐1‐infected individuals on ART. J Leukoc Biol. 2014;96(6):1055‐1063. 25157027 10.1189/jlb.3A0414-232RR PMC4226794

[71] Ben‐Sasson SZ , Wang K , Cohen J , Paul WE . IL‐1β strikingly enhances antigen‐driven CD4 and CD8 T‐cell responses. Cold Spring Harb Symp Quant Biol. 2013;78:117‐124. 24092469 10.1101/sqb.2013.78.021246

[72] Jain A , Song R , Wakeland EK , Pasare C . T cell‐intrinsic IL‐1R signaling licenses effector cytokine production by memory CD4 T cells. Nat Commun. 2018;9:3185. 30093707 10.1038/s41467-018-05489-7 PMC6085393

[73] Van Den Eeckhout B , Tavernier J , Gerlo S . Interleukin‐1 as innate mediator of T cell immunity. Front Immunol. 2021;11:621931. 33584721 10.3389/fimmu.2020.621931 PMC7873566

[74] Vinchi F , Sparla R , Passos ST , et al. Vasculo‐toxic and pro‐inflammatory action of unbound haemoglobin, haem and iron in transfusion‐dependent patients with haemolytic anaemias. Br J Haematol. 2021;193(3):637‐658. 33723861 10.1111/bjh.17361 PMC8252605

[75] Longo F , Gianesin B , Voi V , et al. Italian patients with hemoglobinopathies exhibit a 5‐fold increase in age‐standardized lethality due to SARS‐CoV‐2 infection. Am J Hematol. 2022;97(2):E75‐E78. 34861054 10.1002/ajh.26429 PMC9011434

[76] Carsetti R , Agrati C , Pinto VM , et al. Premature aging of the immune system affects the response to SARS‐CoV‐2 mRNA vaccine in β‐thalassemia: additional dose role. Blood. 2022;140:1735‐1738. 36004936 10.1182/blood.2022017594 PMC9420073

[77] Fattahi S , Khalifehzadeh‐Esfahani Z , Mohammad‐Rezaei M , Mafi S , Jafarinia M . PI3K/Akt/mTOR pathway: a potential target for anti‐SARS‐CoV‐2 therapy. Immunol Res. 2022;70:269‐270. 35107743 10.1007/s12026-022-09268-x PMC8808470

[78] Lechauve C , Keith J , Khandros E , Fowler S , et al. The autophagy‐activating kinase ULK1 mediates clearance of free α‐globin in β‐thalassemia. Sci Transl Med. 2019;11(506):eaav4881. 31434755 10.1126/scitranslmed.aav4881 PMC7441525

