# Supplementary material for: Effects of Sirolimus treatment on patients with β‐Thalassemia: Lymphocyte immunophenotype and biological activity of memory CD4 + and CD8 + T cells
Source: J Cell Mol Med. 2023 Jan 10;27(3):353–64. doi: 10.1111/jcmm.17655 (PMC9889681; doi:10.1111/jcmm.17655)
Supplement: Supplementary file 1 — Data S1 [file JCMM-27-353-s001.docx]

**Effects of Sirolimus treatment on patients with**

**β-Thalassemia: Lymphocyte immunophenotype and biological activity of memory CD4^+^ and CD8^+^ T cells**

**Matteo Zurlo^a^, Francesco Nicoli^b^, Davide Proietto^b^, Beatrice Dallan^b^, Cristina Zuccato^a^, Lucia Carmela Cosenza^a^, Jessica Gasparello^a^, Chiara Papi^a^, Elisabetta d’Aversa^a^, Monica Borgatti^a^, Chiara Scapoli^c^, Alessia Finotti^a^ and Roberto Gambari^a,d^**

^a^Department of Life Sciences and Biotechnology, Section of Biochemistry and Molecular Biology, University of Ferrara, Italy

**^b^** Department of Chemistry, Pharmaceutical and Agricultural Sciences, University of Ferrara, Italy

^c^Department of Life Sciences and Biotechnology, Section of Biology and Evolution, University of Ferrara, Italy

^d^Center ‘Chiara Gemmo and Elio Zago’ for the Research on Thalassemia,

University of Ferrara, Italy

**Corresponding author** Professor Roberto Gambari, Department of Life Sciences and Biotechnology, University of Ferrara, Via Fossato di Mortara n.74, 44121 Ferrara, Italy; Tel: +39-0532-974443; Fax: +39-0532-974500; email: [gam@unife.it](mailto:gam@unife.it).

**SUPPLEMENTARY MATERIAL**

**Supplementary Methods**

**SM1: Bioplex-based Analysis of cytokines, chemokines and growth factors.** Plasma samples isolated from β-Thalassemia patients were obtained by direct centrifugation of the whole blood as described by Zuccato et al. [40].

Proteins were measured using Bio-Plex Human Cytokine 27-plex Assay (Bio-Rad) as suggested by the manufacturer and described in Gasparello et al. [56] and in the Supplementary Materials (SM1). Briefly, an amount of 50 μl of cytokine standards and plasma samples were incubated with 50 μl of anti-cytokine conjugated beads in a 96-well plate. After multiple washing, 25 μl of diluted detection antibody were added to each well and the plate was incubated for 30 min at room temperature with shaking. After washing, 50 μl of streptavidin-phycoerythrin were added, the plate was incubated with shaking at room temperature, washed and read using the Bio-Plex 200 array reader (Bio-Rad). Data were analyzed by the Bio-Plex Manager Software (Bio-Rad) [56].

**SM2. Statistical analysis of plasma levels of cytokines, chemokines and growth factors.**

All the data were presented as mean ± S.D., statistical differences between groups were compared using Wilcoxon test. Statistical differences were considered significant when p< 0.05 (*), highly significant when p< 0.01 (**).

**Supplementary Figures**


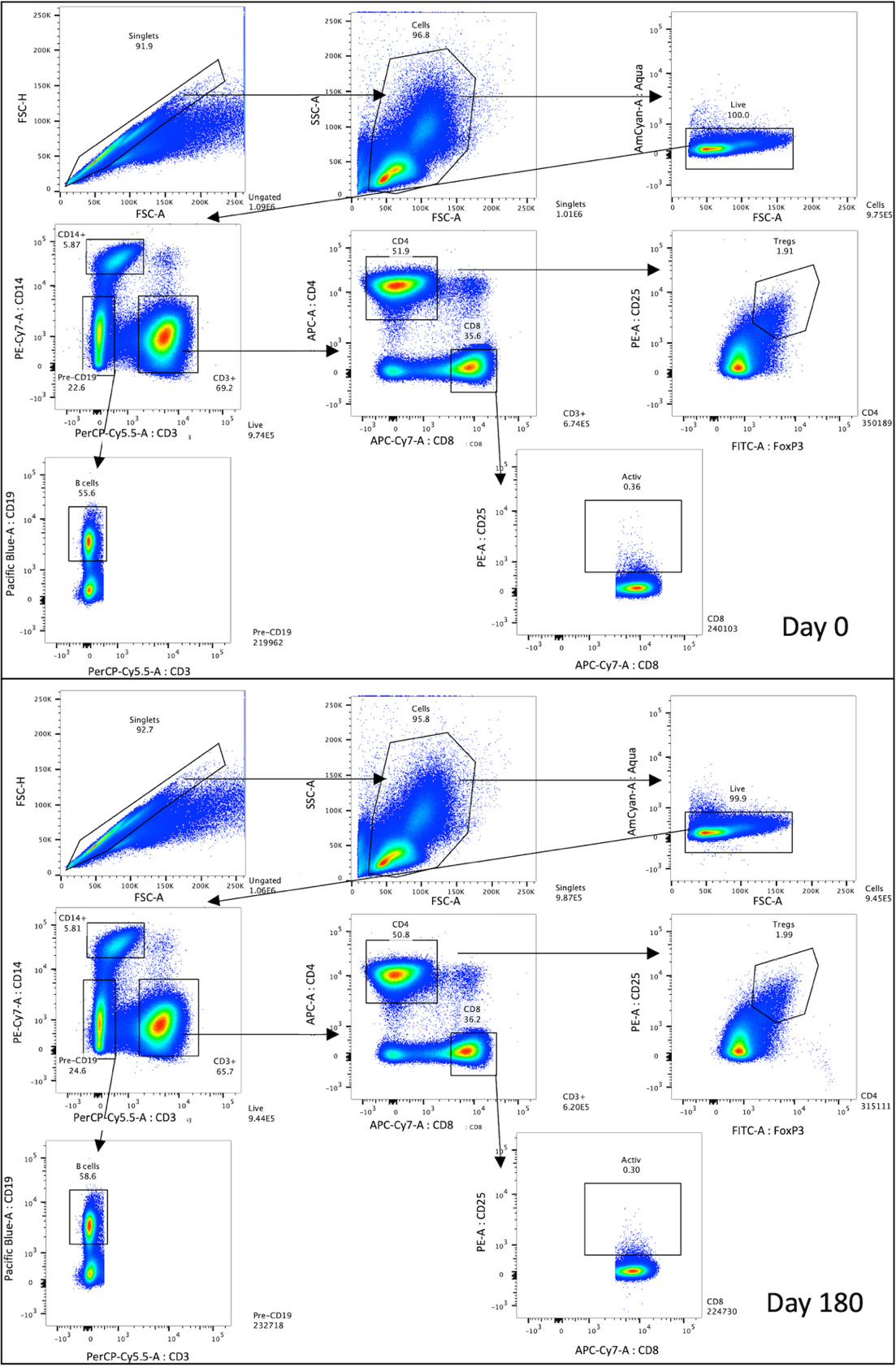


**Figure S1.** Representative Lymphocyte typing data obtained from patient n.14, showing the employed gating strategy. Before starting Sirolimus treatment (upper panel) and after 180 days of treatment (lower panel).


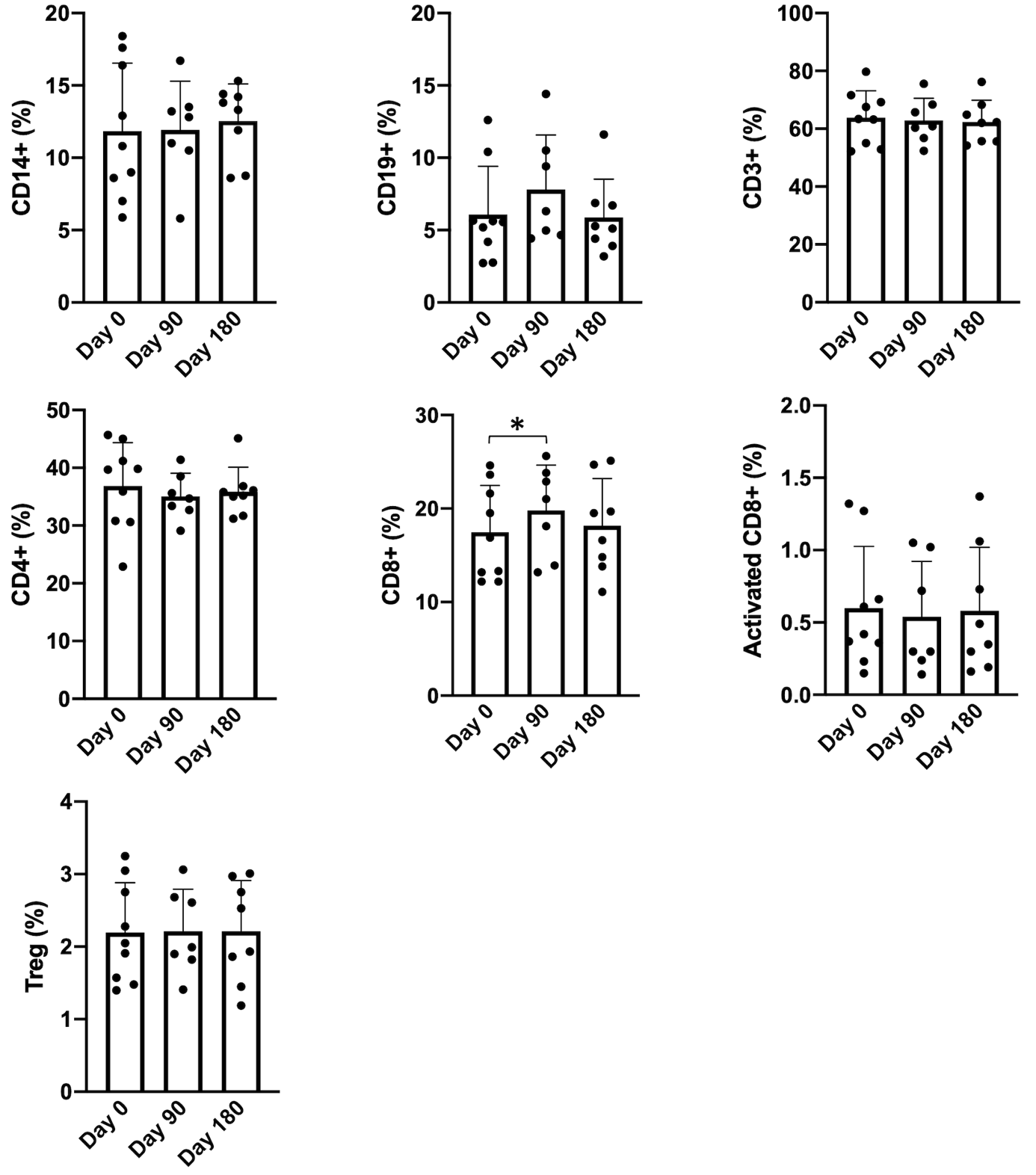


**Figure S2.** Summary representing the mean values of each cellular subset tested by immunophenotyping (Day 0 N=9, Day 90 N=7, Day 180 N=8). *P< 0.05, **P< 0.01. The presented values are expressed as a percentage of positive cells for a given marker with respect to the totality of live cells analyzed, except for activated T lymphocytes, which are respectively expressed as a percentage of CD8^+^ lymphocytes, and Treg lymphocytes, which are expressed as a percentage of CD4^+^ T lymphocytes. This analysis extends and further details the set of data presented in Zuccato et al. [40].


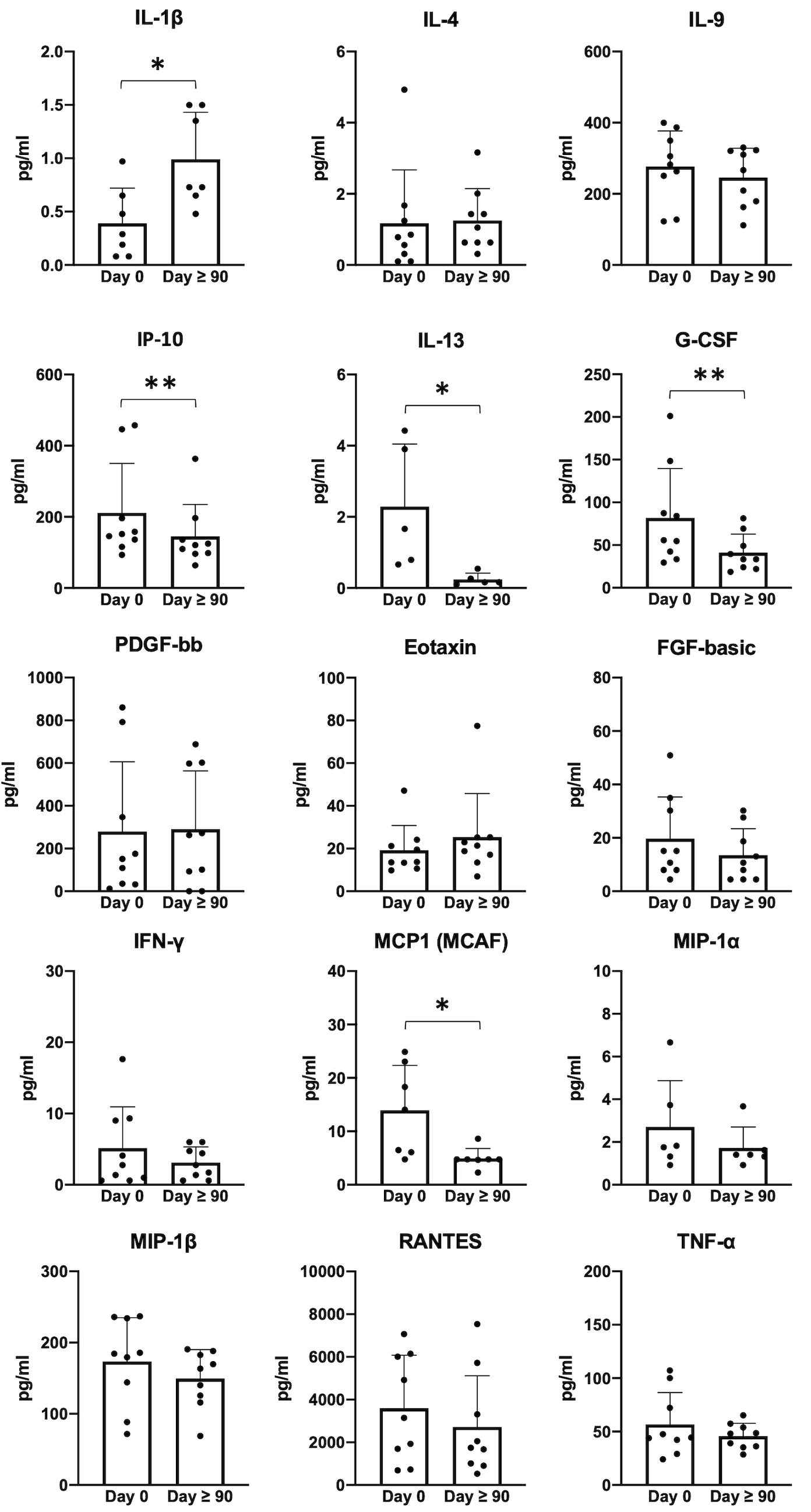


**Figure S3.** Bioplex analysis of plasma samples isolated from β-Thalassemia patients at the beginning (Day 0) or after more than 90 days (Day > 90) treatment with 0.5-2 mg/die Sirolimus as described by Gamberini et al. [39] and by Zuccato et al. [40]. The analysis was conducted using the Bio-Plex Human Cytokine 27-plex Assay (Bio-Rad, Hercules, CA, USA). The secreted proteins for which the majority of patient samples contained <OOR (out of range) values were excluded from the comparative analysis. The best statistically significant values were obtained for IL-1β, IP-10, IL-13, G-CSP and MCP1(MCAF) (discussed in the main text).

**References**

The reference list is available in the Main Text.
